# Improving the Delivery of SOD1 Antisense Oligonucleotides to Motor Neurons Using Calcium Phosphate-Lipid Nanoparticles

**DOI:** 10.3389/fnins.2017.00476

**Published:** 2017-08-30

**Authors:** Liyu Chen, Clare Watson, Marco Morsch, Nicholas J. Cole, Roger S. Chung, Darren N. Saunders, Justin J. Yerbury, Kara L. Vine

**Affiliations:** ^1^Illawarra Health and Medical Research Institute Wollongong, NSW, Australia; ^2^Science Medicine and Health Faculty, Centre for Medical and Molecular Bioscience, School of Biological Sciences, University of Wollongong Wollongong, NSW, Australia; ^3^Department of Biomedical Sciences, Faculty of Medicine and Health Sciences, Macquarie University Sydney, NSW, Australia; ^4^School of Medical Sciences, University of New South Wales Sydney, NSW, Australia

**Keywords:** amyotrophic lateral sclerosis, motor neurone disease, drug delivery, calcium phosphate nanoparticle, antisense oligonucleotide, SOD1, therapeutic intervention, zebrafish

## Abstract

Amyotrophic Lateral Sclerosis (ALS) is a fatal neurodegenerative disease affecting the upper and lower motor neurons in the motor cortex and spinal cord. Abnormal accumulation of mutant superoxide dismutase I (SOD1) in motor neurons is a pathological hallmark of some forms of the disease. We have shown that the orderly progression of the disease may be explained by misfolded SOD1 cell-to-cell propagation, which is reliant upon its active endogenous synthesis. Reducing the levels of SOD1 is therefore a promising therapeutic approach. Antisense oligonucleotides (ASOs) can efficiently silence proteins with gain-of-function mutations. However, naked ASOs have a short circulation half-life and are unable to cross the blood brain barrier (BBB) warranting the use of a drug carrier for effective delivery. In this study, calcium phosphate lipid coated nanoparticles (CaP-lipid NPs) were developed for delivery of SOD1 ASO to motor neurons. The most promising nanoparticle formulation (Ca/P ratio of 100:1), had a uniform spherical core–shell morphology with an average size of 30 nm, and surface charge (ζ-potential) of −4.86 mV. The encapsulation efficiency of ASO was 48% and stability studies found the particle to be stable over a period of 20 days. *In vitro* experiments demonstrated that the negatively charged ASO-loaded CaP-lipid NPs could effectively deliver SOD1-targeted ASO into a mouse motor neuron-like cell line (NSC-34) through endocytosis and significantly down-regulated SOD1 expression in HEK293 cells. The CaP-lipid NPs exhibited a pH-dependant dissociation, suggesting that that the acidification of lysosomes is the likely mechanism responsible for facilitating intracellular ASO release. To demonstrate tissue specific delivery and localization of these NPs we performed *in vivo* microinjections into zebrafish. Successful delivery of these NPs was confirmed for the zebrafish brain, the blood stream, and the spinal cord. These results suggest that CaP-lipid NPs could be an effective and safe delivery system for the improved delivery of SOD1 ASOs to motor neurons. Further *in vivo* evaluation in transgenic mouse models of SOD1 ALS are therefore warranted.

## Introduction

Amyotrophic lateral sclerosis (ALS) is an incurable neurodegenerative disease that is associated with protein misfolding and aggregation. Accumulation of toxic proteinaceous aggregates in the upper and lower motor neurons of the motor cortex and spinal cord over time is associated with motor neuron death, leading to loss of muscle control, muscle atrophy, and invariably death (Brettschneider et al., 2014). The causes of most ALS cases remain undefined, however ~5–10% are inherited (Majoor-Krakauer et al., 2003). Mutations in genes known to cause ALS are growing and include, F*US/TLS, VAPB, TARDBP, OPTN, VCP, SQSTM1, UBQLN2, CCNF, PFN1, MATR3*, and hexanucleotide repeat expansions in *C9ORF72* (Renton et al., 2014). Due to its early discovery in 1993, the best-studied mutation associated with the familial form of ALS (fALS) is in the gene encoding copper/zinc superoxide dismutase (Cu/Zn SOD, SOD1) (Rosen et al., 1993).

SOD1 is a 32-kDa homodimeric metalloenzyme, found primarily in the nucleus, plasma membrane, and cytosol. It contains an active site that binds a catalytic copper ion and a structural zinc ion where it serves to catalyze the dismutation of superoxide radical to dioxygen and hydrogen peroxide (Perry et al., 2010). The correctly folded and active form of the SOD1 enzyme is obtained through several post-translational modifications such as the acquisition of zinc and copper ions, disulfide bond formation, and dimerization (Arnesano et al., 2004). However, mutations that lead to the dissociation of metal ions, and reduction of the intramolecular disulfide bond are known to decrease its conformational stability and promote misfolding. It is well accepted that mutations in SOD1 cause ALS through a toxic gain of function rather than a loss of its native function. Over 180 mutations in the *SOD1* gene that impact upon its structural stability have now been identified (http://alsod.iop.kcl.ac.uk/Overview/gene.aspx?gene_id=SOD1).

A common pathological hallmark in SOD1-ALS cases is the abnormal accumulation of mutant SOD1 in motor neurons of the affected nervous tissues. Our group has recently reported that misfolded SOD1 is present in sporadic disease as well as SOD1-linked fALS, and can propagate cell-to-cell in a prion-like fashion potentially contributing to the orderly progression of the disease (Grad et al., 2014). In addition, we have shown that this cell-to-cell transmission of SOD1 protein aggregates is dependent on fluid-phase endocytosis pathways, primarily via stimulated macropinocytosis (Zeineddine et al., 2015), and relies on active SOD1 synthesis and its presence in a form readily incorporated into aggregates in recipient cells (Grad et al., 2014). Reducing the levels of monomeric and/or misfolded SOD1 is therefore a promising therapeutic target for familial and potentially some sporadic SOD1-related forms of ALS.

Considering the key role copper plays in regulating SOD1 protein stability and function and that low levels have been associated with SOD1 misfolding (Bourassa et al., 2014), a novel ALS treatment based on the concept of copper supplementation to motor neurons has recently been suggested. In the SOD1^G93A^ transgenic mouse model of ALS, the administration of CuATSM, a compound capable of transporting copper into the brain, extended the lives of ALS rodents by 18 months (Soon et al., 2011). Furthermore, restoring copper homeostasis with CuATSM treatment was found to rescue neurons from their symptomatic stage. The effect of CuATSM in patients with familial SOD1-ALS is currently being examined in a Phase I clinical trial (NCT03136809). Another approach currently under clinical investigation involves intraventricular or intrathecal delivery of antisense oligonucleotide against SOD1 for patients with SOD1 fALS (Smith et al., 2006). In a Phase I, randomized, first-in-man study, Miller et al. reported a reduction of SOD1 concentrations in brain tissue, and that this correlates with reduced SOD1 in the CSF (Miller et al., 2013). Although these data strongly support use of SOD1 antisense oligonucleotide as a therapy, the route of administration is invasive by nature and poses a greater risk of post-surgery complications, particularly for repeat dosing.

**Figure 1:**
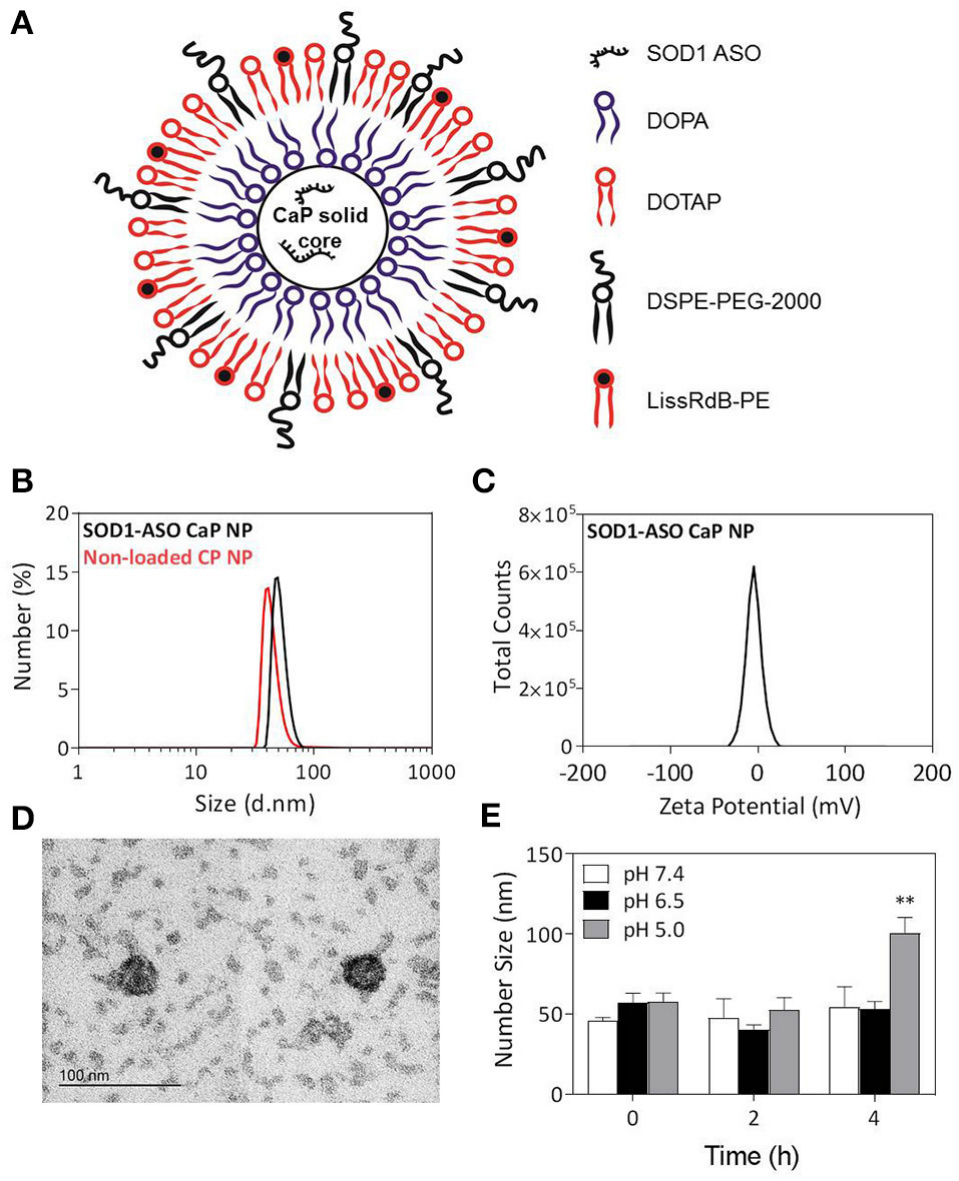
Physical properties of SOD1-ASO-loaded CaP-lipid NPs with a Ca/P ratio 100:1. Schematic representation of SOD1-ASO loaded CaP-lipid NP formulation **(A)**, particle size (d.nm) of SOD1-ASO loaded (black) and non-loaded (red) CaP-lipid NPs determined by DLS **(B)**, zeta potential of SOD1-ASO CaP-lipid NP **(C)**, transmission electron micrograph (TEM) of SOD1-ASO CaP-lipid NPs visualized by negative staining **(D)**, pH-sensitivity as indicated by change in number size (d.nm) of CaP-lipid NPs in PBS at pH 7.4, 6.5, and 5.0 following incubation at 37°C for up to 4 h **(E)**. ^**^P < 0.01.

Nanomedicine is a rapidly growing field that has produced several drug delivery vehicles, such as lipid and polymeric-based nanoparticles, able to deliver genes, and other therapeutics to tissues including the brain and spinal cord (Soppimath et al., 2001; Vieira and Gamarra, 2016). Mechanistically, a drug poorly distributed to the brain can be loaded on/into a nanocarrier system which interacts with the endothelial cells at the blood brain barrier (BBB) to produce higher drug concentrations in brain parenchyma. These nanocarriers can be further modified with targeting moieties to preferentially bind to putative receptors or transporters expressed at the BBB for enhanced CNS selectivity, permeability, and drug trafficking. For example, liposomes surface functionalized with apoE fragments, folate, transferrin or anti-transferrin receptor (anti-TfR) antibody have been shown to cross the BBB by transcytosis and deliver genes to the nervous system, including neurons, with little toxicity (Mc Carthy et al., 2015). In ALS, only one preclinical study has used nanoparticles (glutathione functionalized liposomes) to transport a drug across the BBB, and increase its availability in the CNS, but disappointingly failed to deliver cargo into neurons (Evans et al., 2014). Herein we report the manufacture and biophysical characterization of solid core calcium phosphate lipid nanoparticles (CaP-lipid NPs) that can encapsulate antisense oligonucleotide directed to SOD1 (Figure 1A). We also describe for the first time nanoparticle distribution in the brain, spinal cord, and blood circulation of zebrafish, a powerful experimental vertebrate model for studying ALS. We propose that CaP-lipid NPs can increase the successful delivery of oligonucleotide in ALS.

## Materials and methods

### Reagents and chemicals

Cholesterol, dioleoylphosphatydic acid (DOPA), 1,2-dioleoyl-3-trimethylammonium-propane chloride salt (DOTAP), 1,2-dipalmitoyl-sn-glycero-3-phosphoethanolamine-N-(lissaminerhodamine B sulfonyl) (ammonium salt) (LissRdB-DSPE), and 1,2-distearoryl-sn-glycero-3-phosphoethanolamine-N-[methoxy-(polyethyleneglycol-2000) ammonium salt (DSPE-PEG) were purchased from Avanti Polar Lipids, Inc. (Alabaster, AL). All other chemicals were obtained from Sigma-Aldrich (St. Louis, MO) without further purification. SOD1 antisense oligonucleotide (target sequence 5′-CCG TCG CCC TTC AGC ACG CA-3′) (Patent US 20120214865 A1) and a scrambled negative control oligonucleotide (target sequence 5'-GCC AGC CTA CGA CTC CGC TC-3′) were synthesized by GeneWorks. The scrambled control sequence was generated using an online tool provided by GenScript, USA (https://www.genscript.com/ssl-bin/app/scramble).

### Preparation of oligonucleotide-loaded CaP lipid nanoparticles

Non-loaded or SOD1 antisense oligonucleotide (ASO)-loaded calcium phosphate lipid nanoparticles (CaP-lipid NPs) were prepared as described by Li et al. (Schneider et al., 2012) with minor modifications. The anionic lipid coating CaP core were firstly prepared by a water-in-oil micro-emulsion method. Briefly, 300 μL of 500 mM CaCl_2_ and 50 μL of 2 mg/mL SOD1 antisense oligonucleotide were added in 15 mL cyclohexane/Igepal CO-520 (70/30 v/v) solution to form a very well dispersed water-in-oil reverse micro-emulsion. To form the phosphate phase, 300 μL of 5 mM Na_2_HPO_4_ (pH = 9.0), 50 μL of 2 mg/mL SOD1 antisense oligonucleotide and 200 μL of 20 mg/mL dioleoylphosphatydicacid (DOPA) in chloroform were dispersed in another 15 mL cyclohexane/Igepal solution. Then, the phosphate phase was added to the calcium phase in a dropwise manner. The solution was stirred for 20 min to form the CaP cores encapsulating SOD1 ASO. The micro-emulsion was divided equally in to two 50 mL falcon tubes for the purpose of centrifugation. Absolute ethanol (15 mL) was added to each tube before centrifugation at 9,000 × g for 30 min in Heraeus Megafuge X3R (Thermo Scientific, USA). The pellet of CaP cores was washed and rinsed with ethanol 3 times centrifuging as above each time. The pellet was resuspended in 1 mL chloroform and stored in a small glass vial for subsequently lipid coating. The final CaP lipid-coated nanoparticles were prepared by the thin film hydration method. Briefly, to 500 μL of CaP cores, 50 μL 10 mM DOTAP/Cholesterol (1:1) and 50 μL 3 mM DSPE-PEG-2000 were added. For cellular uptake studies, LissRdB-DSPE (12.5 μL of a 16 mM stock solution) was also added. The solution was transferred to a round bottom flask and the chloroform was evaporated off using a rotary evaporator (Büchi, Switzerland). Tris-HCl buffer (1 mL; 5 mM; pH 7.4) was then added into the round bottom flask to rehydrate the lipid film, and the solution was gently sonicated for 5 min. The final CaP-lipid NPs were sterile filtered (0.22 μm) and stored at 4°C for subsequent characterization and biological evaluation.

### Particle characterization

Zeta (ζ) potential of CaP-lipid NPs was measured using a Malvern Zetasizer ZS (Malvern, CA), while size was determined by dynamic light scattering (DLS) using a Malvern Zetasizer APS instrument (Malvern, CA). NanoSight (LM14, NanoSight, UK) was used to calculate particle concentration. Specifically, particles were diluted to between 10^8^–10^10^ particles/mL particles (~1:500 dilution of particles in Milli-Q water) before observing via conventional optical microscope equipped with a CCD camera at room temperature. The particle motion (20–100 particles per field of view) was recorded at 30 frames for 60 s. Particle concentration was then calculated via the NanoSight software (NTA 2.3, UK). To visualize the morphology of CaP-lipid NPs we used transmission electron microscopy (TEM). The nanoparticle suspension was dropped onto a 200 mesh carbon coated copper grid and stained with 1% phosphotungstic acid (PTA). The stained suspension was washed using Milli-Q water and dried at room temperature before observing on a transmission electron microscope (TEM; Gantan, Inc.). TEM was carried out using a JEOL 2010 instrument operating at 200 kV. Bright field images were captured with an objective aperture inserted to enhance diffraction contrast. Images were recorded using a Gatan Orius camera coupled to Gatan's Digital Micrograph software. Oligonucleotide loading was determined using the propidium iodide (PI) staining method whereby the final SOD1 ASO-loaded CaP-lipid NPs were dissolved in an equal volume of lysis buffer (2 mM EDTA and 0.05% Triton X-100 in pH 7.8 Tris buffer) and heated at 65°C for 20 min to release the entrapped SOD1 ASO for fluorometric measurement. To each 100 μL sample, PI (1 μL; 1 μg/μL) was added and fluorescence (RFLU) was measured using a FLUOstar plate reader (BMG Labtech, Germany) (Ex = 540 nm; Em = 620 nm). The amount of SOD1 ASO loaded into CaP-lipid NPs was then determined by interpolation of RFLU from a SOD1 ASO standard curve. The stability of SOD1-loaded CaP-lipid NPs was assessed via the change in particle size (number peak area) over time measured by DLS. Briefly, 100 μL SOD1 CaP-lipid NP suspension was incubated at 4°C under sterile conditions, and the peak area of particles measured at the following time points 0, 4, 8, 12, 16, 20, 24, and 28 days. For pH-responsive studies, the particles were diluted 10 times (v/v) in PBS (pH 7.4, 6.5, or 5), and incubated for 2 and 4 h at 37°C with constant shanking at 200 RPM. The change in particle size (number peak area) was recorded by DLS. The corresponding anti-dilution ability was also detected to ensure that the CaP-NPs retain their size upon dilution in complete DMEM media supplemented with 10% FCS.

### Cell lines and culture conditions

The mouse motor neuron-like cell line (NSC-34), a hybrid cell line produced by fusion of neuroblastoma with mouse motor neuron-enriched primary spinal cord cell (Cashman et al., 1992), was routinely cultured in DMEM/F12 supplemented with 10% (v/v) FBS. The human embryonic kidney (HEK293) cell line, which has been used extensively as an expression tool to study recombinant proteins, was cultured in the same media. Cells were maintained in an incubator at 37°C under a humidified atmosphere containing 5% (v/v) CO_2_. When 80% confluence was reached, cells were detached by incubation with 5 mM Trypsin-EDTA and harvested after centrifugation in a Heraeus Megafuge 1.0 (Thermo Scientific, USA) at 1,200 rpm for 5 min at RT. Cells were resuspended in media, and viable cells counted using a hemocytometer and trypan blue staining. Cells were confirmed free of Mycoplasma contamination.

### *In vitro* cellular uptake

NSC-34 cells were seeded at 75,000 cells/cm^2^ into eight well-chamber slides, and incubated overnight at 37°C before the addition of sterile filtered (0.22 μm) non-loaded CaP-lipid NPs containing LissRdB-DSPE (1:5 or 1:10 v/v). Cells were then incubated for a further 30, 60, or 90 min at 37°C to measure cellular uptake over time. Media was removed and cells were washed in chilled 1 × PBS before being imaged using a Leica TCS SPII laser scanning confocal microscope (Heidelberg, Germany). Internalization was quantified by calculating the mean fluorescence per cell from five images per treatment using ImageJ (Schneider et al., 2012). This represents a minimum of 100 cells analyzed per treatment. To confirm the mechanism of particle uptake, LysoTracker Green DND-26 (Life Technologies, Mulgrave, VIC) was used to stain for acidic compartments, lysosomes and endosomes. NSC-34 cells were incubated at 37°C with Lysotracker Green DND-26 for 30 min, before the addition of LissRdB-DSPE CaP-lipid NPs and incubation for a further 30 min. Imaging of live cells was carried out as described above.

### *In vitro* transfection and gene silencing

HEK293 cells were seeded in 6-well plates at a density of 20 × 10^4^ cells/well. After reaching 60% confluency, the cells were treated with SOD1-loaded CaP-lipid NPs, and incubated at 37°C for 72 h. Control treatments included non-loaded CaP-lipid NPs, SOD1 ASO or scrambled negative control with Lipofectamine® 2,000 Transfection Reagent (ThermoFisher Scientific, Rockford, IL, USA) and non-loaded CaP-lipid NPs with SOD1 ASO free in solution. Each treatment delivered an equivalent amount of ASO. Cells were washed twice with chilled PBS and harvested with 0.05% Trypsin-EDTA. Cell lysates were collected after adding RIPA lysis buffer (25 mM pH7.6 Tris-HCl, 150 mM NaCl, 1% Triton X-100, 1% Na-deoxycholate and 1% SDS) supplemented with HALT Protease Inhibitor Cocktail (1% protease inhibitor and 1% EDTA; ThermoFisher Scientific). The final protein extracts were obtained after centrifuging (12,000 × g, 5 min) the aspirated cell lysates. Total protein concentration in the lysate was determined by Bio-RAD Assay according to manufacturer's instructions using a protein assay kit (Micro BCA™ Protein Assay Kit, Pierce).

Total protein (15 μg) was loaded on a pre-cast sodium dodecyl sulfate polyacrylamide gel (Bio-Rad, Gladesville, NSW) and electrophoresed at 150 mV for 45 min. The proteins were transferred to a pure nitrocellulose blotting membrane at 1.0A and 25 V for 30 min using the Bio-Rad Trans-Blot Turbo system. The membrane was blocked with 5% skim milk in TBST on a horizontal shaker overnight at 4°C. The membrane was incubated with 1:1000 sheep polyclonal antibody to SOD1 (ab8866; Abcam, Melbourne, VIC) for 1 h at 37°C on an orbital shaker followed by incubation with HRP-conjugated donkey anti-sheep IgG secondary antibody (AB324P; Merck Millipore, Bayswater, VIC) for 1.5 h at room temperature on a horizontal shaker. Finally, SuperSignal West Pico Chemiluminescent Substrate (ThermoFisher Scientific) was added to the membrane and it was exposed using an Amersham 600RGB Imager (GE).

### *In vivo* delivery and visualization of CaP nanoparticles

Zebrafish (Danio rerio) at an age of 4–6 days post fertilization (dpf) were used for the injection studies. Experimental protocols were approved by Macquarie University Animal Ethics Committee (using zebrafish to understand how the central nervous system responds to neuronal stress and death caused by neurodegenerative diseases; protocol no. 2015/033). Zebrafish were maintained at 28°C in a 13 h light and 11 h dark cycle. Embryos were collected by natural spawning and raised at 28.5°C in E3 and PTU (1-phenyl-2-thiourea) solution according to standard protocols (Westerfield, 2000; Morsch et al., 2015). For *in vivo* studies, sterile filtered non ASO-loaded CaP-lipid NPs containing LissRdB-DSPE (stock, 1:5 or 1:10 v/v) were used for injections into the zebrafish. Borosilicate capillary glass needles with filament (WPI Inc.) were pulled to a resistance between 2 and 7 MΩ and filled with 4 μL of non-loaded CaP-lipid NPs containing LissRdB injection solution. A microinjection apparatus (WPI Inc.; Picospritzer II, General Valve Corporation) was used under control of a stereo dissection microscope (Leica, M165FC) to deliver the NPs into the area of interest (~1–2 nL per injection). To ensure precise injection anesthetized zebrafish were mounted into low-melting agarose (Fisher-Scientific) at a concentration of 1–1.5% according to established protocols (Morsch et al., 2017). Control injections were performed with the same dilution of free LissRdB and fish were imaged under the same imaging conditions. Imaging was performed at various time-points (immediately after injections, 2 and 24 h post injection) using a compound microscope (Leica DMI300b), a structured illumination microscope (Zeiss ApoTome), or a confocal microscope (Leica SP5) as described previously (Morsch et al., 2015). To confirm localization of NPs in the zebrafish spinal cord and brain, injections were performed into transgenic lines with fluorescent labeling of neurons [motor neurons: Tg(-3mnx1:TagBFP); pan-neuronal: Tg(isl1:GFP)] (Don et al., 2017). Localization in the brain and vasculature was confirmed with transgenic lines labeling CNS astrocytes [Tg(GFAP:EGFP)] or the entire vasculature [Tg(fli1a:EGFP)], respectively.

### Statistical analysis

Data are presented as the mean ± standard deviation based on triplicate values from two or more independent experiments (*n* > 6). Student's *t*-test or one-way analysis of variance (ANOVA) using a Tukey's multiple comparisons *post-test* were used to determine statistical significance (GraphPad Prism 6 software). *P* < 0.05 was considered statistically significant.

## Results

### Preparation and characterization of SOD1-ASO CaP-lipid nanoparticles

Previous studies have demonstrated that the stoichiometry of calcium to phosphate can influence the size, encapsulation efficiency, and polydispersity of CaP nanoparticles (Olton et al., 2007; Tang et al., 2015). In this study, the physical properties of SOD1-ASO CaP-lipid NPs were optimized through variation of the Ca/P ratio from 20:1 to 100:1, by changing either the concentration of CaCl_2_ or Na_2_HPO_4_. Particle size decreased with increasing concentration of CaCl_2_ (100–500 mM) (Table 1). Increasing the Ca/P ratio to 200:1 and 400:1, with the corresponding concentration of CaCl_2_ at 1000 and 2000 mM, respectively, lead to poor nanoparticle re-hydration (data not shown). An inverse trend in particle size was obtained when increasing the concentration of Na_2_HPO_4_ from 5 mM to 25 mM, whilst keeping the concentration of CaCl_2_ at 500 mM (Table 1). The encapsulation efficiency (EE) of ASO was also influenced by the concentration of CaCl_2_ and Na_2_HPO_4_. We used an encapsulation method for SOD1-ASO analogous to that reported by Tang et al. where half of the ASO was mixed with the calcium and the phosphate solutions to yield a significantly greater EE than if the ASO was loaded into the calcium or phosphate phases alone (Tang et al., 2015) (Figure S1). The EE decreased from 69.09 ± 2.7 to 47.87 ± 5.6% when increasing the Ca/P ratio from 20:1 to 100:1, respectively. However, the difference in EE between 50:1 and 100:1 Ca/P formulations was not significant as determined by one-way ANOVA (Table 1). Conversely, the EE of the SOD1-ASO CaP-NPs peaked at 47.87 ± 2.8% and displayed a declining trend as the content of Na_2_HPO_4_ increased, when the concentration of CaCl_2_ was fixed at 500 mM. The EE appeared to be independent of particle concentration. All ASO-loaded particles were slightly negatively charged, likely due to the anionic lipid DOPA in the inner lipid layer and the DSPE-PEG-2000 in the outer lipid layer which has a net negative charge at pH 7.4. Encapsulation of SOD1 ASO into CaP-lipid NPs with a Ca/P ratio of 100:1 significantly decreased the zeta potential from −03 ± 0.03 to −4.9 ± 0.1 mV. Free SOD1 ASO was −50.1 ± 1.8 mV (Figure S2). In light of the optimal particle size, concentration and charge that would be required for crossing the BBB in future studies, we chose the nanoparticle formulation with a Ca/P ratio of 100:1 for all subsequent experiments. Full physicochemical data of this formulation including size, zeta potential, and morphology using TEM is presented in Figures 1B–D.

**Table 1:** Particle size, Zeta (ζ)-potential, polydispersity index, and encapsulation effiency of SOD1 antisense oligonucleotide calcium phosphate-lipid nanoparticles (SOD1-ASO CaP-lipid NPs) prepared at different CaCl_2_, and Na_2_HPO_4_ concentrations.

**CaCl_2_ (mM)**	**100**	**250**	**500**	**500**	**500**
Na_2_HPO_4_ (mM)	**5**	**5**	**5**	**10**	**25**
CaP Ratio	20/1	50/1	100/1	1/50	1/20
No. Size (nm)	61.0 ± 13.7	52.3 ± 14.7	31.0 ± 2.2	39.9 ± 3.3	50.4 ± 7.6
Zeta Potential (mV)	−6.3 ± 0.2	−7.2 ± 0.2	−4.9 ± 0.1	−6.6 ± 0.51	−6.4 ± 0.25
Polydispersity Index (PDI)	0.2 ± 0.019	0.2 ± 0.005	0.3 ± 0.006	0.2 ± 0.014	0.2 ± 0.007
Encapsulation Effiencicy (%)	69.1 ± 2.7	51.1 ± 2.56	47.9 ± 5.6	34.3 ± 4.15	16.1 ± 2.07
Particle Concentration (per mL)	0.7 E^11^	2.6 E^11^	11.9 E^11^	2.6 E^11^	5.3 E^11^

*Data is presented as the average ± SD (n = 5)*.

### pH sensitivity of CaP-ASO-nanoparticles

The disassembly of CaP-lipid NPs at low pH allows for the release of the encapsulated ASO from acidic compartments into the cytoplasm. The lead CaP-lipid NP formulation (Ca/P ratio of 100:1) displayed pH-dependant dissolution after 4 h (Figure 1E). No significant difference in particle size was observed when the particles were incubated at 37°C in PBS at pH 7.4, and pH 6.5, however, particle size increased sharply to 84.8 nm 4 h post incubation at pH 5, indicating particle disassembly. The results suggest that CaP-lipid NPs exhibit good pH-responsive release of cargo under acidic conditions with sufficient incubation time. Stability studies found the particles to be stable in Tris buffer over a period of 20 days (Figure S3). With respect to the stability of particles upon dilution, which would occur if using a systemic route of administration, the CaP-lipid NPs exhibited a robust anti-dilution effect. No significant change in particle size was detected when CaP-lipid NPs were diluted 1:10 and 1:50 in complete DMEM supplemented with 10% FCS (Figure S4), indicating the as-prepared CaP-lipid NPs are likely to retain colloidal stability in circulation when delivered systemically in future *in vivo* studies.

### CaP-lipid nanoparticle uptake into NSC-34 motor neuron-like cells is time dependent

**Figure 2:**
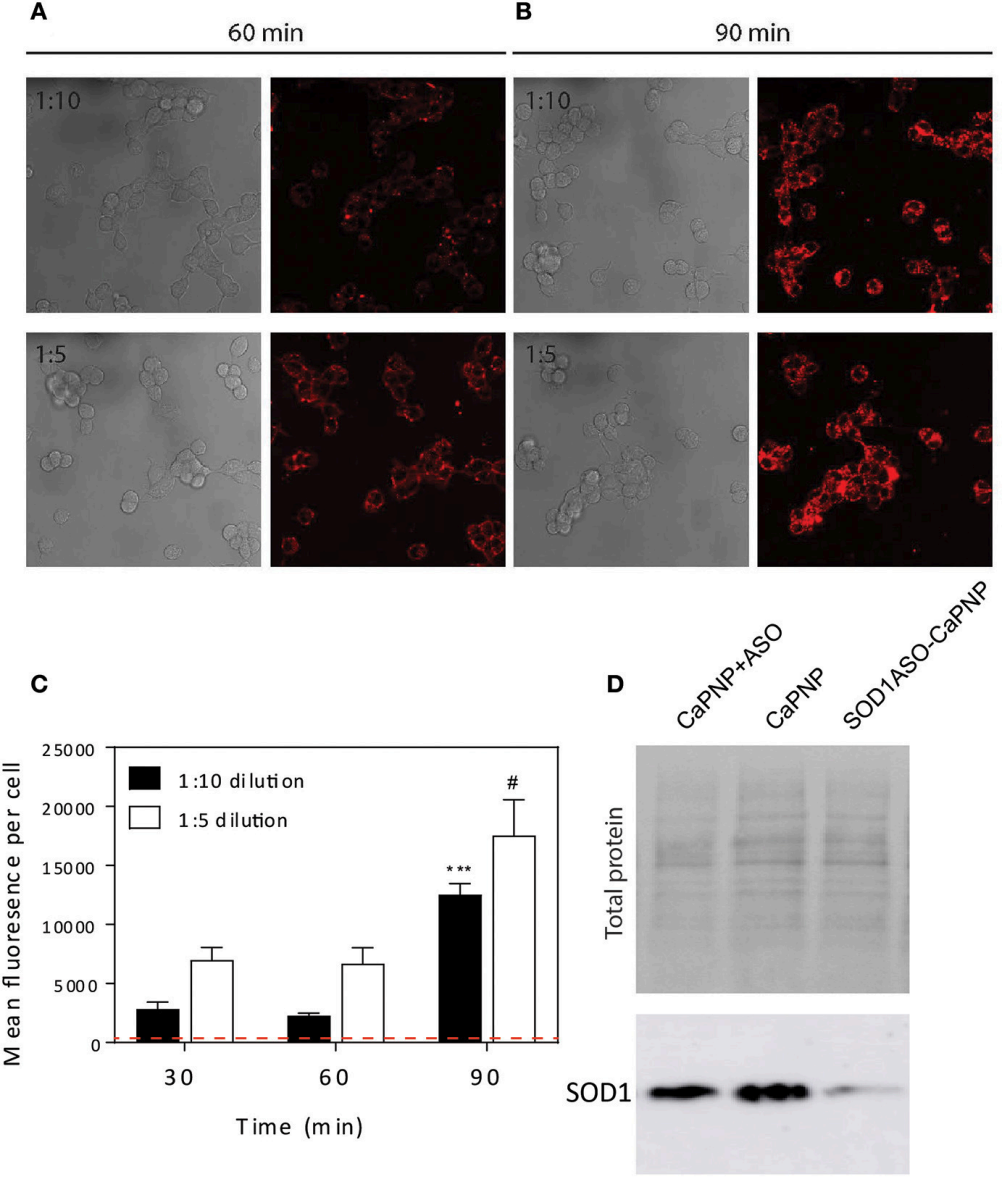
Internalization of CaP-lipid NPs into NSC-34 cells and knockdown of SOD1 protein expression in HEK293 cells. The CaP nanoparticles contained LissRdB-DSPE (Ex = 560 nm; Em = 583 nm) in the outer lipid layer. NSC-34 cells were incubated at 37°C with LissRdB-DSPE CaP-lipid NPs diluted either 1:5 or 1:10 (v/v) for 30, 60 **(A)** and 90 min **(B)**. Images were acquired with a 63 × objective (512 × 512 pixels; physical length 246.03 μm × 246.03 μm). Change in average cell fluorescence over 90 min incubation period **(C)**. Red dotted line represents auto fluorescence of NSC-34 cells for comparison. ^***^P < 0.001 and #*P* < 0.05 signifies significant difference compared to respective 30 min data. HEK293 cells were treated with SOD1-ASO CaP-lipid NPs for 72 h at 37°C (Lane 3). Control treatments included non-loaded CaP-lipid NPs (Lane 2) and non-loaded CaP-lipid NPs with SOD1-ASO free in solution (Lane 1). Controls using Lipofectamine 2000 Transfection Reagent with SOD1-ASO or scrambled negative SOD1 oligonucleotide are not shown here. The change in SOD1 protein expression (16 kDa) was visualized sing SuperSignal West Pico Chemiluminescent Substrate and exposed using an Amersham 600RGB Imager **(D)**.

It is well known that CaP NPs in the size range of 25–40 nm can be internalized by various cell lines (Li et al., 2012; Tang et al., 2015). Here we sought to determine if our CaP-lipid NPs could be internalized by motor neuron-like cells. Nanoparticles containing LissRdB-DSPE were added to the media of NSC-34 motor neuron-like cells at two dilutions (1:5 or 1:10, total particle concentration 2.69 × 10^11^.mL^−1^ and 1.35 × 10^11^.mL^−1^, respectively). The fluorescent properties of LissRdB-DSPE allowed us to monitor uptake of the nanoparticles in live cells. Following incubation for 30–60 min, confocal microscopy confirmed close association of our nanoparticles with NSC-34 cells (Figures 2A,C), indicating that nanoparticles were entering the cytoplasm, and accumulating in florescent foci, but were absent in the nucleus (Figure 2). There was minimal auto fluorescence from NSC-34 cells (Figure 2C, Figure S5), allowing us to easily distinguish, and quantify cellular uptake of fluorescent CaP-lipid NPs. After 30 min, a proportion of the CaP-lipid NP signal was co-localized to acidic compartments, late endosomes, and lysosomes (Figure S6). The uptake of nanoparticles was dependent upon time, with a significant increase in fluorescence after 90 min of incubation (Figures 2B,C).

### ASO delivered by CaP nanoparticles decreases SOD1 levels *in vitro*

SOD1 ASO infused directly into brains via the lateral ventricle has been used in preclinical models, including rats, and Rhesus monkeys (Smith et al., 2006). It has also been shown to significantly reduce SOD1 levels in human fibroblasts after transfection (Smith et al., 2006). Here we compared the *in vitro* efficacy of SOD1 knockdown by free ASO to our SOD1-ASO-CaP-lipid NPs. Due to the ASO being directed toward human SOD1, our knockdown experiments were performed in the HEK293t cell line as they are human derived. Cells were incubated with either empty CaP-lipid NPs, empty CaP-lipid NPs + free (un-encapsulated) SOD1 ASO, or SOD1-ASO-CaP-lipid NPs. Compared to empty nanoparticles alone, there was a small decrease in SOD1 levels after incubation with free SOD1 ASO (Figure 2D). Incubation with SOD1 ASO encapsulated within CaP-lipid NPs resulted in an 8× reduction in SOD1 levels, compared with free SOD1 ASO (Figure 2D). While naked ASO can enter cells inefficiently and interact with mRNA after infusion into the CNS (Smith et al., 2006), our data shows a significantly increased SOD1 knockdown through our CaP-lipid NP drug delivery system.

### *In vivo* distribution of CaP-lipid nanoparticles in zebrafish

**Figure 3:**
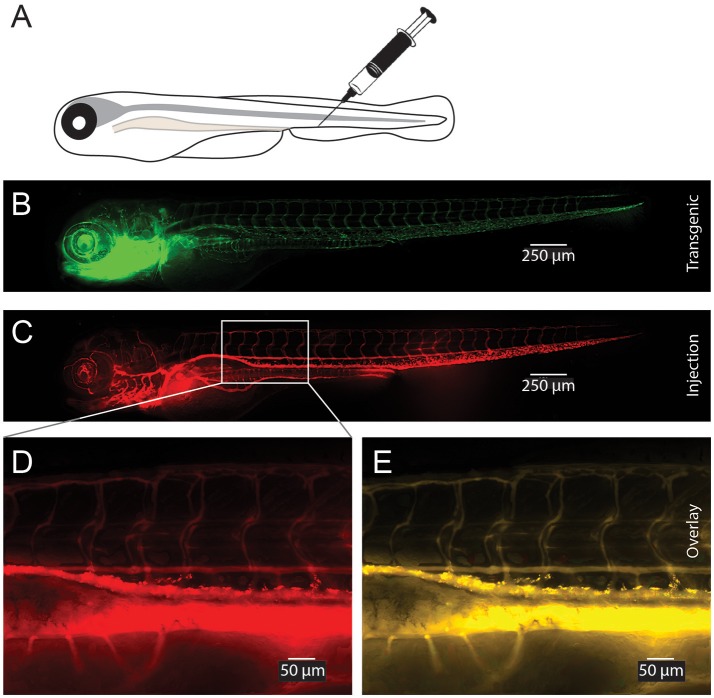
Visualization of CaP-lipid NPs after tail vein injection in transgenic zebrafish. Sterile filtered non ASO-loaded CaP-lipid NPs containing LissRdB-DSPE (stock, 1:5 or 1:10 v/v) was injected into the vein of zebrafish expressing a green fluorescent reporter in their vasculature (Tg(fli1a:EGFP)). Schematic illustration of the route of injection **(A)**. Expression of EGFP in transgenic fish highlighting the blood vessels **(B)**. Visualization of CaP-lipid NP in 6-day-old transgenic zebrafish 2 h after injection **(C)**. Zoomed image of the treated zebrafish from area indicated by white box, showing distribution and accumulation of CaP-lipid NP in and around blood vessels **(D)**. Overlay of transgenic EGFP expression and CaP-lipid NP distribution **(E)**.

**Figure 4:**
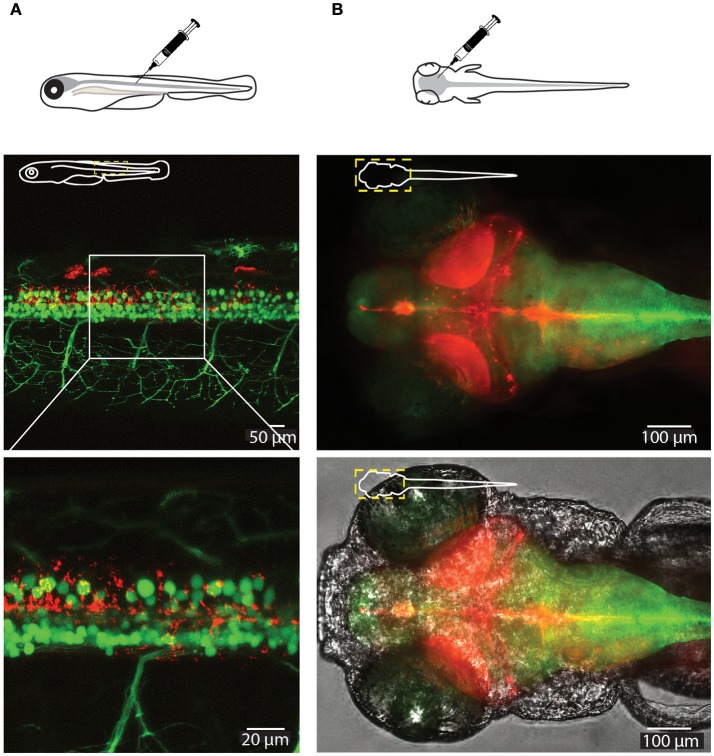
Visualization of CaP-lipid NPs and neurons in the spinal cord and brain of a 6-dpf zebrafish. Sterile filtered non ASO-loaded CaP-lipid NPs containing LissRdB-DSPE (red) were micro injected into the zebrafish spinal cord **(A)** and brain **(B)**. **(A)** Visualization of CaP- lipid NPs containing LissRdB-DSPE (red; injections) within the spinal cord neurons (green; transgenic expression) of a 6-day-old transgenic zebrafish. Zoomed image of the treated zebrafish from area indicated by white box, showing distribution of the CaP-lipid NPs within GFP-expressing spinal cord neurons. **(B)** Expression of brain-injected CaP-lipid NPs (red) in a transgenic zebrafish expressing astrocyte-specific GFP [green; Tg(GFAP:GFP)] to highlight the brain-specific delivery of these particles. The bottom image overlays the bright-field channel for better visualization of the CNS. The schematic inserts in panels depict the orientation of the fish and outline the presented area.

To test the delivery of the nanoparticles *in vivo* we microinjected small volumes (1–2 nL) of empty CaP-lipid NPs into living zebrafish larvae. Our CaP-lipid NPs were able to diffuse throughout the brain, and spinal cord after direct injection. Injections into the brain, the bloodstream and the spinal cord resulted in characteristic accumulation of speckle-like structures (Figures 3, 4) compared to the free fluorescent reporter (Figure S7). Retro-orbital injections into the zebrafish eye (Pugach et al., 2009) or injections into the posterior cardinal vein, representing systemic routes of delivery, resulted in distinctive uptake of fluorescent NPs into the bloodstream within minutes after injection (Figures 3A–C). The particles were detectable with high fluorescence emission within the vasculature of the zebrafish 2 h post administration (Video S1). After injection into the zebrafish brain we observed rapid NP accumulation along the ventricle and throughout the whole brain (Figure 4B). Injections directly into the spinal cord of the living zebrafish showed accumulation of the CaP-lipid NPs around neurons and occasionally co-localized with neuronal labeling within 2 h of injection (Figure 4A). Altogether, our *in vivo* application via microinjections revealed an efficient delivery method for these nanoparticles in order to test their potential in disease modification in the future.

## Discussion

Gene therapies (mostly antisense oligonucleotides; ASO) are in use for a range of disorders where increasing or decreasing the production of a specific gene is of therapeutic benefit. A number of gene therapies are already in clinical use for various cancers, Gaucher's disease and Rheumatoid Arthritis reviewed in Stein and Castanotto (2017). While there are no approved gene therapy products on the market for ALS, multiple oligonucleotide-based compounds are under development for the treatment of brain disorders by direct delivery inside the BBB. Notably, the first new central nervous system (CNS)-targeted oligonucleotide-based drug (nusinersen/Spinraza) was approved by the US Food and Drug Administration (FDA) in late 2016 for spinal muscular atrophy (SMA) in pediatric and adult patients and several compounds are beginning to be trialed in a range of other neurodegenerative diseases (O'Connor and Boulis, 2015; Khorkova and Wahlestedt, 2017). So far there have been three gene therapy clinical trials in ALS based on either expressing growth factors (VEGF and HGF) or ASOs to knockdown SOD1 reviewed in Scarrott et al. (2015). To date, clinical trials in ALS have identified that gene therapies are tolerable, and safe; however more work is needed to understand the specific advantages and disadvantages of the delivery methods. For example, while SOD1 ASOs hold great promise, they are unable to cross the BBB and as such rely on intraventricular or intrathecal infusion, an invasive surgical procedure whereby ASO is delivered directly to the CNS. Furthermore, initial clinical trials involving SOD1 ASO infusion did not reduce SOD1 levels in the CNS at the dose trialed in humans (Scarrott et al., 2015). In this study, we encapsulated SOD1 ASO in an optimized formulation of CaP-lipid NPs and examined their biophysical properties, effectiveness of ASO delivery *in vitro* and biodistribution *in vivo*.

The stoichiometry of calcium to phosphate has been reported to influence the size, encapsulation efficiency (EE), and polydispersity of CaP nanoparticles (Olton et al., 2007; Tang et al., 2015). In this study, the EE decreased as the ratio of Na_2_HPO_4_ increased. This is likely due to the fact that the ionic crosslinking of negatively charged phosphates of the ASO with positively charged Ca^2+^ ions would decrease if more ${\text{PO}}_{4}^{3-}$ ions were present during the co-precipitation step (Truong-Le et al., 1999; Uskoković and Uskoković, 2011). In contrast, the size of the particles varied such that the largest particles were observed when prepared using the highest, and lowest ratios of Ca/P, a phenomenon similarly described by Tang et al. (2015). The smallest particles were made using a Ca/P ratio of 100:1. In general, for optimal cell uptake via receptor mediated endocytosis, particles should be smaller than the clatherin coated vesicle size (i.e., <200 nm) (Traub, 2009). Of particular interest here is that small particle size is always preferred for brain-drug delivery due to the rigorous restriction of the BBB (Silva et al., 2017). In addition, Lockman et al. highlights the importance of nanoparticle surface charge (ζ-potential) on the integrity, and crossing of the BBB, with neutral and anionic nanoparticles preferable over cationic particles as colloidal drug carriers to the brain (Lockman et al., 2004). Taken together, the properties of nanoparticle size, charge, encapsulation efficiency, and polydispersity index (PDI) led us to select the SOD1-ASO CaP-lipid NPs with Ca/P molar ratio of 100:1 (500 mM CaCl_2_ and 5 mM Na_2_HPO_4_) as our lead formulation.

Previous work has shown that CaP NPs in the size range of 25–40 nm can be efficiently internalized by various cell lines (Li et al., 2012; Tang et al., 2015). Our results suggest that our CaP-lipid NPs (31.0 ± 2.2 nm) are rapidly taken up by motor neuron-like NSC-34 cells in a time and concentration dependent manner. At least partial overlap with a lysotracker dye suggests trafficking of the CaP-lipid NPs to acidic compartments, likely late endosomes and lysosomes (Liu et al., 2014). Our optimized CaP-lipid NPs were stable in solutions of neutral pH for at least 20 days, but broke down at pH 5 in as little as 4 h. Once a CaP NP has dissolved it is thought that the released calcium and phosphate ions cause changes in osmotic pressure across the lysosomal membrane causing rupture of the vesicle (Neumann et al., 2009) and release of the ASO. This principle has for many years been used to transfect mammalian cells with plasmid DNA. In this study, the efficiency of ASO delivery in cell culture was increased through CaP-lipid NP encapsulation, and resulted in significant reduction in SOD1 protein expression. This is likely due to the pH sensitivity, lysosomal membrane rupture, and cytoplasmic delivery of ASO. Importantly, CaP transfection methods have been successfully used on primary neurons for decades (Dudek et al., 1997), suggesting that CaP-lipid NPs are a viable system for the delivery of DNA/RNA to neurons in the brain. CaP has primarily been used as a transfection agent because it is inexpensive and easy to use, biocompatible, bioactive, and can alter the osmotic properties of the lysosome. While there have been reports that transfection with CaP can cause large-scale lysosome rupture and subsequent necrosis (Liu et al., 2014), packaging CaP into nanoparticles suppresses this toxicity (Neumann et al., 2009), making it safe for intracellular delivery. Moreover, CaP NPs are thought to have a high loading capacity when compared to other inorganic particles, and provide several advantages over viral delivery systems, including low immunogenicity, and the ability to switch off or modify therapeutic dose.

Some genetic lesions associated with ALS result in toxic gain-of-function and have been the traditional targets for genetic therapeutic strategies (Foust et al., 2013). In particular, the largest known genetic causes of ALS, mutations in SOD1, and C9ORF72, are both thought to be gain of toxic function mechanisms, making genetic knockdown a relevant, and important avenue for research. Yet this strategy has achieved only modest outcomes to date in preclinical development. For example, knockdown of SOD1 using viral delivered shRNA in the SOD1^G93A^ ALS mouse model found only 8% of neurons had been successfully targeted in 3 week old mice (Foust et al., 2013). Non-viral vectors are therefore gaining traction as alternative delivery systems due to their potential safety advantages, ease of manufacture, customization, BBB targeting potential, and ability to deliver all nucleic acid varieties [reviewed in Niidome and Huang (2002) and Pack et al. (2005)]. The BBB presents both a physical and electrostatic barrier to limit brain permeation of therapeutics. Previous work has demonstrated that nanoparticles can overcome the physical barrier and deliver genes to the nervous system including neurons, by utilizing transcytosis pathways when functionalized with various ligands including apoE fragments, folate, transferrin or anti-transferrin receptor (anti-TfR) antibodies (Mc Carthy et al., 2015). This surface functionalization could also be applied to CaP-lipid NPs and has been successfully demonstrated after i.v. administration in rodent models (Shi and Pardridge, 2000; Shi et al., 2001; da Cruz et al., 2005; Re et al., 2011; Qiao et al., 2012). In ALS preclinical models, the only nanoparticle so far used has been glutathione functionalized liposomes that targeted methylprednisolone (a glucocorticoid) across the BBB, and increased its availability in the CNS (Evans et al., 2014). Two of the main reasons for a lack of effective neuroprotective therapy for ALS can be attributed to a number of key challenges including ineffective targeting and delivery of therapeutic agents specifically to the diseased CNS site and/or cell type of interest (Scarrott et al., 2015). However, targeted delivery across the BBB and concomitant internalization by cells of the brain, in particular motor neurons using such nanotechnology has so far not been developed for ALS.

Zebrafish are emerging as a powerful model organism to study neurodegenerative diseases. This is primarily due to features such as experimentally feasible (short) developmental life time, external fertilization, well established methods of transgenesis, and importantly transparent bodies that allow imaging of the living nervous system (Westerfield, 2000; Morsch et al., 2017). In the context of ALS, zebrafish models have been used to study familial forms, and recapitulate some aspects of ALS pathology, including loss of motor neurons (Hogan et al., 2017), making them a useful system for gene therapy testing. In addition, the ability to generate compound transgenic zebrafish permits strategies to track and follow different cell types and different pathogenic proteins concomitantly, making them a valuable addition to current model systems for study of ALS. Previous work has shown the utility of the fluorescently labeled cell populations and simultaneous delivery of therapeutic cargo encapsulated in fluorescent nanoparticles (Fenaroli et al., 2014). The work presented here demonstrates that our CaP-lipid NPs circulate freely within the bloodstream following systemic delivery in an experimental zebrafish model and were detectable immediately after and up to 29 h post injection. This prolonged circulation time is owed to the hydrophilic nature of PEG within our formulation. PEGylation of particles is well known to create a protective water shell around the particle thereby protecting it against enzymatic degradation, decreasing clearance by the mononuclear phagocyte system and retarding renal clearance, increasing the residence time in the bloodstream (Suk et al., 2016). Finally, our CaP-lipid NPs were able to diffuse throughout the brain and spinal cord after direct injection, demonstrating the usefulness of zebrafish in the study of nanoparticle delivery of therapeutic cargo in ALS. It will be interesting to test the efficacy of our ASO encapsulated CaP-lipid NPs in zebrafish and mammalian models of SOD1 ALS in the future. Taken together, our data suggest that CaP-lipid NPs establish a useful tool to increase gene therapy delivery, and thus efficacy in ALS.

## Author contributions

JY, KV, DS, MM, LC, CW, and RC contributed to the conception or design of the work and/or purchased consumables. JY, KV, CW, LC, and MM acquired, analyzed and interpreted data. JY, KV, DS, MM, LC, CW, and RC drafted the work and/or revised it critically for important intellectual content. All Authors have approved the final version to be published and agree to be accountable for all aspects of the work.

### Conflict of interest statement

The authors declare that the research was conducted in the absence of any commercial or financial relationships that could be construed as a potential conflict of interest.
