# Supplementary material for: Improving the Delivery of SOD1 Antisense Oligonucleotides to Motor Neurons Using Calcium Phosphate-Lipid Nanoparticles
Source: Front Neurosci. 2017 Aug 30;11:476. doi: 10.3389/fnins.2017.00476 (PMC5582160; doi:10.3389/fnins.2017.00476)
Supplement: Supplementary file 2 [file Presentation1.PDF]

frontiers

Supplementary Material

Improving The Delivery Of SOD1 Antisense Oligonucleotides To Motor Neurons Using Calcium Phosphate -Lipid Nanoparticles

L. Chen, C. Watson, M. Morsch, N.J. Cole, R. Chung, D. Saunders, J.J Yerbury and K.L. Vine*

*** Correspondence: Dr Kara Lea Vine**: kara@uow.edu.au

**Contents**	**Page**
**Figure S1**: Optimization of the encapsulation of SOD1 ASO in CaP-lipid NPs	**S2**
**Figure S2**: Comparison of the zeta potential of SOD1 ASO-loaded and non-loaded CaP-lipid NPs	**S2**
**Figure S3**: Stability of CaP-lipid NPs over time	**S3**
**Figure S4**: Anti-dilution effect of CaP-lipid NPs	**S3**
**Figure S5**: Auto fluorescence of NSC-34 cells	**S4**
**Figure S6**: CaP-lipid NPs are internalized into NSC-34 cells and partially co-localize with acidic compartments	**S4**
**Figure S7:** Visualization of control LissRdB injections in zebrafish	**S5**

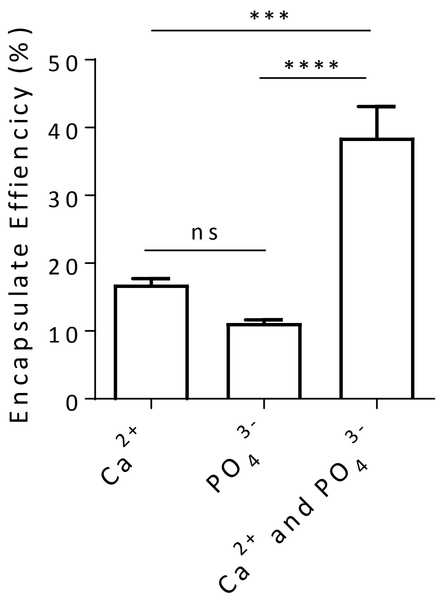

Figure S1. Encapsulation efficiency of SOD1 ASO in CaP-lipid NPs. ASO was added in either the Ca2^{+}, PO_4^{3-} or Ca^{2+} and PO_4^{3-} phase. Data is the mean of triplicates ± SD. NS; not significant, *** P<0.001 and **** P<0.0001.

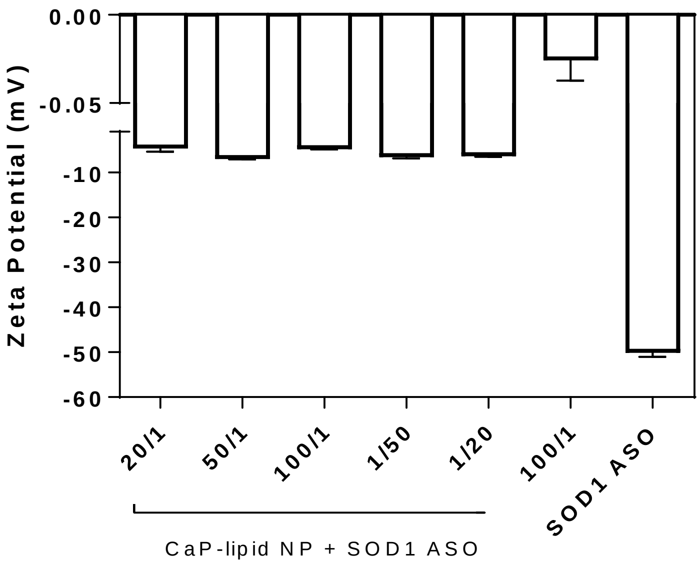

Figure S2. Comparison of the zeta potential (mV) of SOD1 ASO-loaded CaP-lipid NPs with varying Ca/P ratios to non-loaded CaP-lipid NPs (Ca/P 100:1) and free SOD1 ASO.

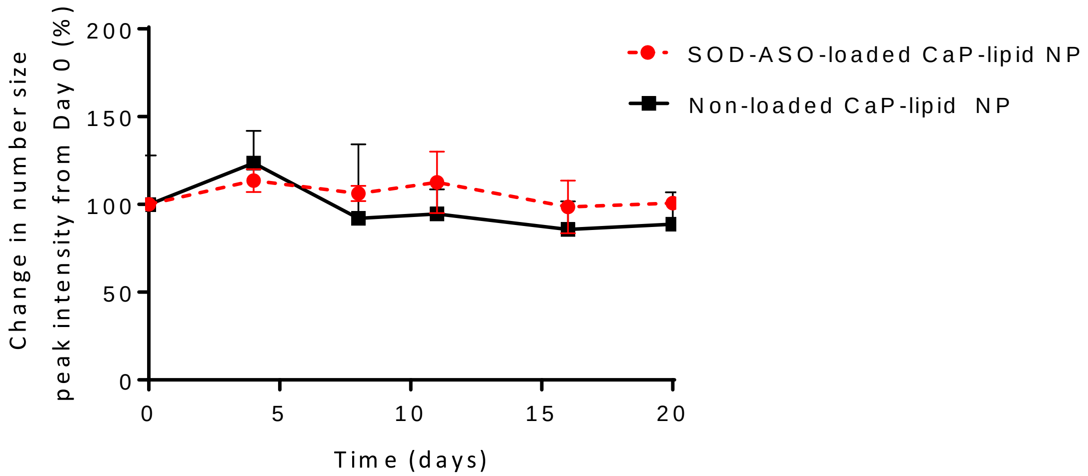

Figure S3. Stability of CaP-lipid NPs over time. The stability of non-loaded calcium phosphate lipid nanoparticles (CaP-lipid NPs) and SOD1-ASO-loaded CaP lipid NPs as prepared in Tris-HCl buffer was measured by DLS over 20 days. No significant change in particle size, measured as the change in peak intensity from the initial measurement, was observed by two-way ANOVA.

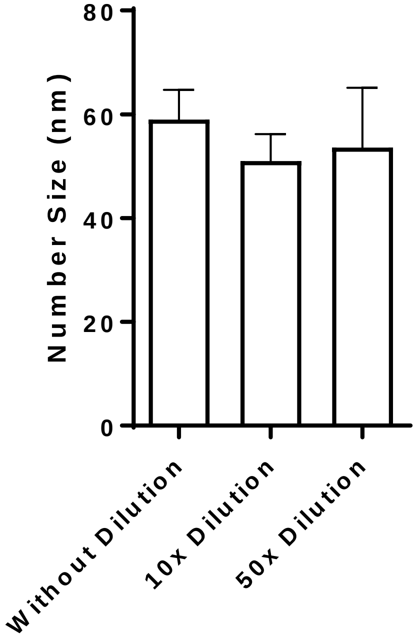

Figure S4. Anti-dilution effect of 100/1 CaP-lipid NPs. The number particle size of non-loaded CaP lipid NPs in DMEM/F12 media supplemented with 10% FCS with 10 and 50 times dilution (v/v). No significant difference was observed between treatments by one-way ANOVA.

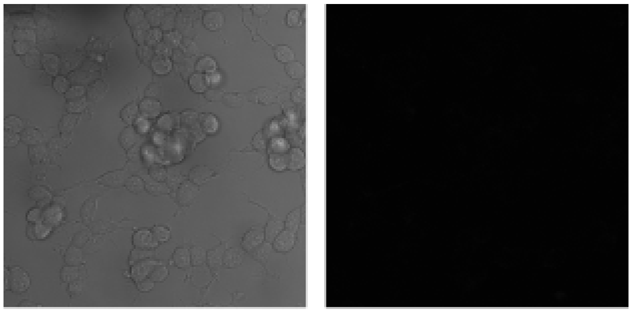

Figure S5. Auto fluorescence of NSC-34 cells. Prior to incubation of LissRdB-DSPE CaP-lipid NPs with NSC-34 cells, cells were imaged to determine background auto fluorescence levels. Images were acquired with a 63 × objective (512 × 512 pixels; physical length 246.03 µm × 246.03 µm).

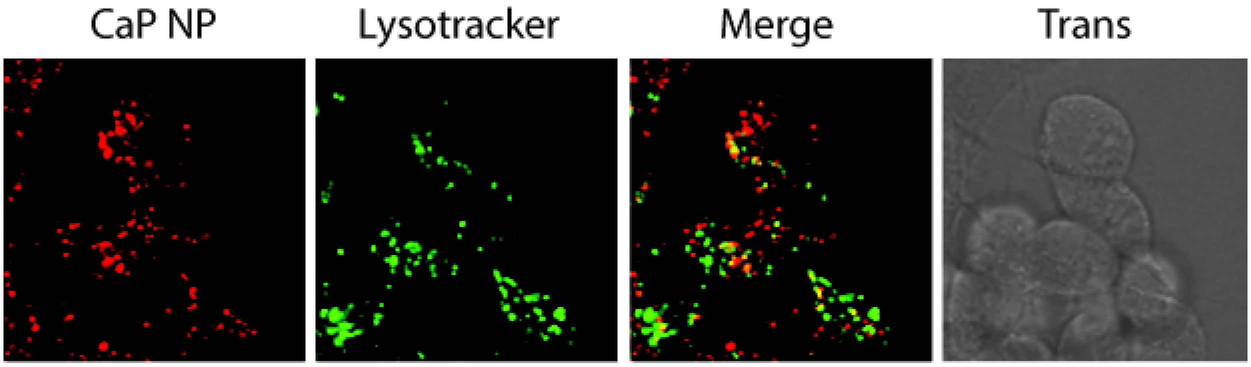

Figure S6. CaP –lipid NPs are internalized into NSC-34 cells and partially co-localize with acidic compartments. The CaP –lipid NPs contained LissRdB-DSPE (Ex =560 nm; Em=583 nm) in the outer lipid layer (red). Lysotracker Green DND-26 was used to label acidic compartments. NSC-34 cells were incubated at 37°C with Lysotracker Green DND-26 for 30 min, before addition of LissRdB-DSPE CaP-lipid NPs for a further 30 min. Yellow areas in the merged image indicate co–localization.

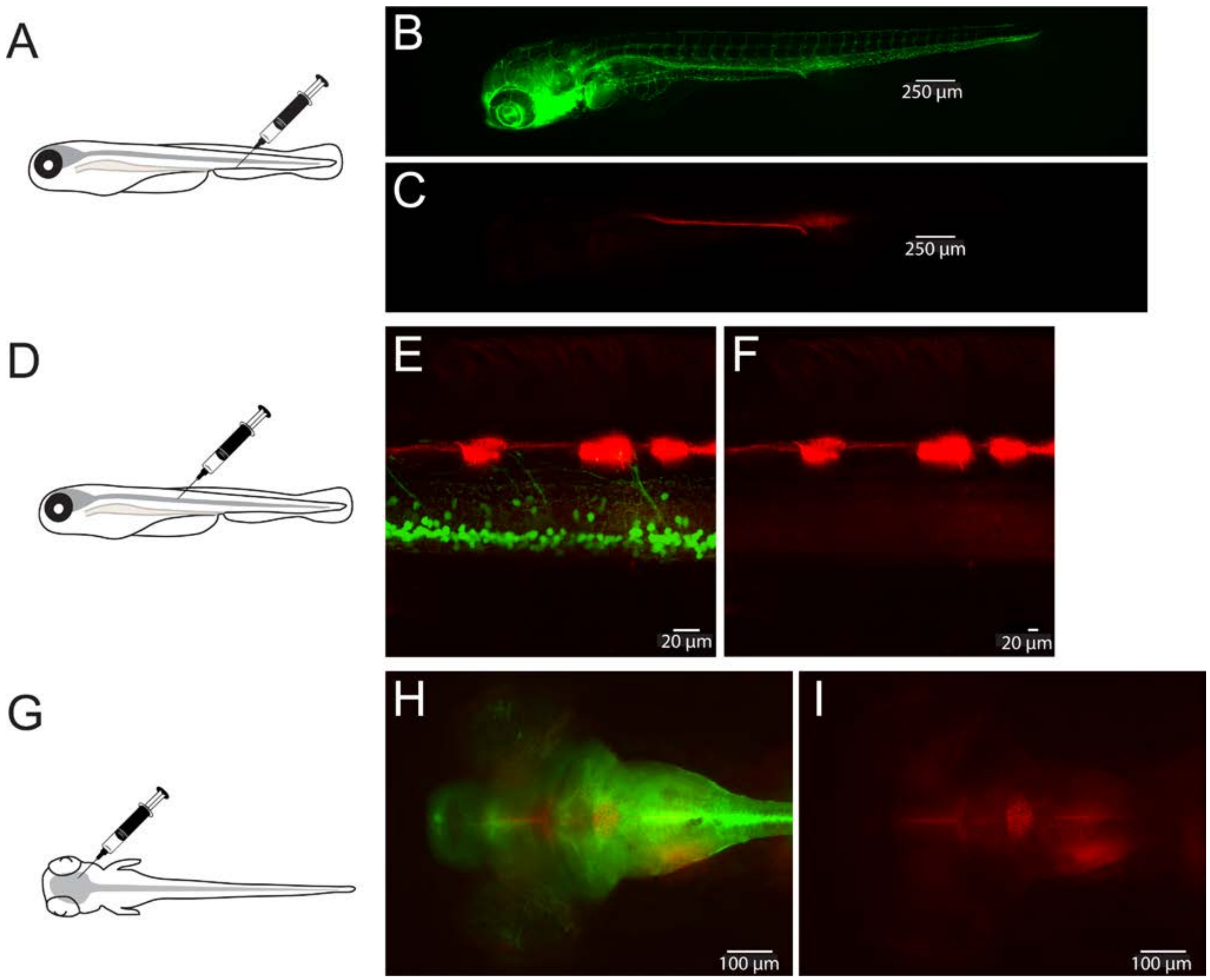

Figure S7. Visualization of control LissRdB injections in the zebrafish. LissRdB was injected into the vasculature (A-C), the spinal cord (D-F) and the brain (G-I) to compare distribution and brightness to CaP-lipid NPs containing LissRdB-DSPE. (A) Schematic representation of zebrafish and vein injection. (B) Transgenic zebrafish expressing EGFP (green) in the vasculature highlighting blood vessels. (C) Minimal expression of control-LissRdB throughout the vessels two hours after injection. (D) Schematic representation of zebrafish and spinal cord injection. (E) Minimal expression of control-LissRdB throughout the neurons of the spinal cord (green) two hours after injection. (F) Control-LissRdB (red) channel only. (G) Schematic representation of zebrafish and brain injection. (H) Minimal expression of control-LissRdB throughout the brain of a transgenic zebrafish (green) two hours after injection. (F) Control-LissRdB (red) channel only.
